# Snake Cathelicidin Derived Peptide Inhibits Zika Virus Infection

**DOI:** 10.3389/fmicb.2020.01871

**Published:** 2020-08-04

**Authors:** Meichen Xing, Mengyao Ji, Jingmei Hu, Tengyu Zhu, Yaoyao Chen, Xuewei Bai, James Mwangi, Guoxiang Mo, Ren Lai, Lin Jin

**Affiliations:** ^1^College of Life Sciences, Nanjing Agricultural University, Nanjing, China; ^2^Key Laboratory of Animal Models and Human Disease Mechanisms, Kunming Institute of Zoology, Chinese Academy of Sciences, Kunming, China; ^3^Sino-African Joint Research Center, Kunming Institute of Zoology, Chinese Academy of Sciences, Kunming, China; ^4^Key Laboratory of Bioactive Peptides of Yunnan Province, Kunming Institute of Zoology, Chinese Academy of Sciences, Kunming, China; ^5^Center for Biosafety Mega-Science, Chinese Academy of Sciences, Wuhan, China; ^6^KIZ-CUHK Joint Laboratory of Bioresources and Molecular Research in Common Diseases, Kunming Institute of Zoology, Chinese Academy of Sciences, Kunming, China; ^7^Institute for Drug Discovery and Development, Chinese Academy of Sciences, Shanghai, China

**Keywords:** Zika virus, host defense peptide, antiviral mechanism, cathelicidin-BF, AXL receptor

## Abstract

Zika virus (ZIKV) is a mosquito-borne virus belonging to the genus *Flavivirus* and has reemerged in recent years with epidemic potential. ZIKV infection may result in severe syndromes such as neurological complications and microcephaly in newborns. Therefore, ZIKV has become a global public health threat and currently there is no approved specific drug for its treatment. Animal venoms are important resources of novel drugs. Cathelicidin-BF (BF-30) is a defensive peptide identified from *Bungarus fasciatus* snake venom and has been shown to be an excellent template for applicable peptide design. In this study, we found that ZY13, one of the peptidic analogs of BF-30, inhibits ZIKV infection *in vitro* and *in vivo*. Mechanistic studies revealed that ZY13 can directly inactivate ZIKV and reduce the production of infectious virions. Further studies also indicated that administration of ZY13 strengthen the host antiviral immunity via AXL-SOCS (suppressor of cytokine signaling protein) pathway. Additionally, the results of mouse experiment suggest that ZY13 efficiently restrict ZIKV infection and improve the growth defects of ZIKV-infected mouse pups. Together, our findings not only demonstrate that ZY13 might be a candidate for anti-ZIKV drug, but also indicated the importance of animal venom peptides as templates for antivirals development.

## Introduction

Zika virus (ZIKV) is a single-stranded RNA virus and predominantly transmitted by the widely distributed Aedes mosquitoes in nature (Barrows et al., 2018). In the past decade, the epidemic potential of ZIKV likely increased with its emergence in the Pacific, Americas, and Asia (Baud et al., 2017). ZIKV can be classified into the African lineage and the Asian lineage and the latter is the recent epidemic strain (Wang et al., 2016; Beaver et al., 2018; Liu et al., 2019). It is reported that a spontaneous mutation of NS1 protein from a clinical ZIKV isolate of the Asian lineage enhanced the infectivity of ZIKV in *A. aegypti* mosquitoes (Liu et al., 2017). Despite some differences between the two lineages, the epidemic ZIKV seems to have shifted from mild self-limited pathogenesis to a severe neuro-virulent virus (Beaver et al., 2018). However, it should be noted that ZIKV infection can cause severe neurological symptoms in fetuses, neonates, and adults (Baud et al., 2017). Additionally, ZIKV can be detected in humans body fluids and may be transmitted through sexual activities (Tang et al., 2016; Zhang et al., 2016; Allard et al., 2017). Moreover, it has been demonstrated that ZIKV can induce inflammation in the mouse testis and leads to male infertility (Govero et al., 2016; Ma et al., 2016). Therefore, controlling ZIKV epidemics is a daunting challenge.

However, there is no approved specific drug and vaccine for ZIKV (Chaudhuri et al., 2018). Although vaccination is the primary strategy for preventing ZIKV infections, antivirals may also play an important role in controlling ZIKV infection and transmission. After the emergence of ZIKV in Brazil in 2015-2016, more efforts are directed at discovering specific anti-ZIKV drug candidates (Li et al., 2017; Alves et al., 2018; Han and Mesplede, 2018). At present, repurposing approaches have identified many FDA-approved drugs and their anti-ZIKV activities have been tested *in vivo*. BCX4430, 7-deaza-2′-*C*-methyladenosine (7DMA), NITD008 and Ebselen (EBS) had been approved for clinical trials while 7DMA and NITD008 were dismissed recently due to their weak activity or undesirable toxicity (Han and Mesplede, 2018). The good news is that with the increasing understanding about ZIKV infection and host antiviral mechanism, more anti-ZIKV compounds can be discovered. For example, following the discovery that ZIKV induced RNAi-mediated antiviral immunity, the antibiotic enoxacin has been found to prevent ZIKV infection by enhancing RNAi (Xu Y. P. et al., 2019).

In recent years, although the peptide antivirals have gained more attention, there are still a couple of hurdles to surmount before the clinical use (Boto et al., 2018; Bruzzoni-Giovanelli et al., 2018). Currently, much progress has been made in designing artificial anti-ZIKV peptides (Yu et al., 2017; He et al., 2018; Si et al., 2019). On the other hand, natural animal host defense peptides (HDPs) which uniquely evolved to protect the hosts have been shown to have antiviral properties (Shartouny and Jacob, 2018). However, although natural HDPs show excellent therapeutic potential, still, certain factors limit their clinical application (Boto et al., 2018). A better understanding of HDPs has enhanced the development of natural peptides derived analogs with improved pharmacological properties and reduced side effects (Fjell et al., 2011; Jin et al., 2016; Boto et al., 2018).

Cathelicidins are evolutionarily conserved HDPs and play critical roles in the battle against pathogens. Cathelicidin-BF (BF-30), a defensive peptide identified from *Bungarus fasciatus* snake venom, has been shown to have strong activities against *Propionibacterium acnes* and Influenza A virus infections in previous studies (Wang et al., 2011; Xu J. et al., 2019). Our previous work indicated that BF-30 is an excellent template for applicable peptide design (Jin et al., 2016; Xu C. et al., 2019). As previously reported, ZY13 is a peptidic analog derived from BF-30 and has been shown to have therapeutic potential for vaginitis (Jin et al., 2016). Recently, BF-30 and its analogs were approved for phase I-III clinical study (CXHL1700235) to develop vaginal effervescent tablet for the treatment of bacterial vaginosis based on its excellent characteristics. Based on the reported anti-RNA virus activity of BF-30, we assumed that the analogs of BF-30 may have potential antiflavivirus effects. In this study, we found that ZY13 inhibits ZIKV infection by directly inactivating ZIKV particles and strengthening the host antiviral immune responses. Additionally, ZY13 has shown efficiency on ZIKV-infected mouse pups. These findings may provide important strategies for developing animal derived peptides specific antiviral drugs.

## Materials and Methods

### Cell Lines and Viruses

U251 cells (human glioma cell line), C6/36 cells (*A. albopictus* cell line) and Vero cells (African green monkey kidney epithelial cell line) were obtained from Kunming Cell Bank, Kunming Institute of Zoology, Chinese Academy of Sciences. U251 cells were cultured in Dulbecco’s Modified Eagle Medium: Nutrient Mixture F-12 (DMEM/F-12) (Gibco, Waltham, MA, United States) supplemented with 10% fetal bovine serum (FBS), 100 U/ml penicillin and 100 μg/ml streptomycin in 5% CO_2_ at 37°C. Vero cells were cultured in DMEM medium (Gibco, Waltham, MA, United States) with the same supplements in 5% CO_2_ at 37°C. C6/36 cells were cultured in RPMI 1640 medium (Gibco, Waltham, MA, United States) supplemented with 10% fetal bovine serum (FBS), 100 U/ml penicillin and 100 μg/ml streptomycin in 5% CO_2_ at 28°C. ZIKV (SZ01, KU866423.2) and DENV-2 (New Guinea-C, KM204118) were kindly provided by Prof. Cheng-feng Qin (Beijing Institute of Microbiology and Epidemiology) and were propagated in C6/36 cells.

### Mouse Bone Marrow Derived Macrophages (BMDMs) and ZIKV Infection Assay

C57BL/6 and IFN-α/β receptor-deficient mice (*Ifnar*^–/–^) mice were kept under specific pathogen-free conditions in the Animal Resource Center at Kunming Institute of Zoology, Chinese Academy of Sciences. All experiments were conducted in accordance with the guidelines and were approved by the Animal Care and Use Committee, Kunming Institute of Zoology, Chinese Academy of Sciences (SMKX-20190513-01). Bone marrow cells from mice were cultured in DMEM supplemented with 10 ng/ml M-CSF (14-8983-80, eBioscience, Waltham, MA, United States), 10% FBS, 0.1 mM non-essential amino acids, and 100 U/ml penicillin and 100 μg/ml streptomycin in 5% CO_2_ at 37°C for 5 days to generate BMDMs.

For virus infection, BMDMs were infected with ZIKV at 1 MOI with or without ZY13 and Amodiaquine (AQ, A2799, Sigma, Burlington, MA, United States) administration. AQ is an FDA-approved drug and can effectively inhibit ZIKV infection in neural progenitor cells both *in vitro* and in a mouse model (Zhou et al., 2017). Twenty four hours post infection, cells were lysed in TRIzol reagent for RNA isolation and further analysis.

### Peptide Synthesis

Peptide ZY13 (VKRWKKWRWKWKKWV) and a scrambled control peptide Scr-ZY13 (KRWVWKRWVKKWKWK) with C-terminals amidated (-NH_2_) were synthesized by GL Biochem (Shanghai) Ltd. (Shanghai, China) and analyzed by reversed-phase high-performance liquid chromatography (RP-HPLC) and mass spectrometry to confirm their purity greater than 98%.

### Cell Viability Assay

Cell viability was evaluated by conventional 3-(4, 5-dimethyl-2-thiazolyl)-2, 5-diphenyl-2H-tetrazolium bromide (MTT) reduction assays in 96-well plates. After a 24 h treatment by testing sample, MTT was added to each well to a final concentration of 0.5 mg/ml and incubated at 37°C for 4 h. The MTT solution was then removed and dimethyl sulfoxide (DMSO) was added to solubilize the MTT-formazan crystals in living cells. The absorbance of the resulting solution was measured at 570 nm.

### Assays for Antiviral Activity Assessment *in vitro*

U251 cells or Vero cells were infected with ZIKV or DENV-2 at indicated MOI and time with or without ZY13, Scr-ZY13, 50 μM of Suramin (574625, Merck Millipore, Darmstadt, Germany), 20 μM of Tin protoporphyrin IX dichloride (SnPPIX, 0747, Bio-Techne, Minneapolis, MN, United States) and 5 μM of AQ administration (Tan et al., 2017; Han et al., 2018; Neris et al., 2018). SnPPIX was found to inactivate ZIKV by targeting the viral envelope whereas suramin was found to inhibit ZIKV attachment and particals release (Albulescu et al., 2017; Neris et al., 2018). The final concentration of the control compounds used in this study was based on previous studies and preliminary experiments. For ZIKV infection, cells were seeded in 12-well plates (2 × 10^5^/well) and incubated with ZIKV in serum- and antibiotic-free cell culture medium for 1h with or without ZY13 and Scr-ZY13. Then the cells were washed and the supernatants were replaced with medium containing 2% FBS with or without ZY13 and Scr-ZY13. After incubation for 24 h, cells were lysed in TRIzol reagent for RNA isolation or fixed in 4% paraformaldehyde for immunofluorescence staining. The supernatants of the cells were also collected.

To determine the ZY13 mediated ZIKV inactivation, peptides at various concentration were added to ZIKV (1 × 10^6^ PFU/ml) followed by incubation at 37°C for 1, 2, and 4 h. The peptide and ZIKV mixtures were then diluted 200 times in DMEM for further infectivity analysis. Considering 200 times dilution cannot remove peptides from the mixture, the equivalent amounts of ZY13 without pre-incubation were applied as control.

To determine whether ZY13 affects ZIKV attachment, assays were performed by pre-incubating U251 cells with ZIKV, ZIKV and peptides or ZIKV and Suramin at 4°C for 1 h. After adsorption, the cells were washed three times with DMEM and the attached ZIKV was detected by qRT-PCR.

### qRT-PCR Analysis and Plaque-Forming Assay

Total RNA was isolated from cells and tissues by using TRIzol reagent (Invitrogen, Waltham, MA, United States). cDNA was reverse-transcribed by using random primer and M-MLV reverse transcriptase (M1705, Promega, Madison, WI, United States). Real-time qRT-PCR was performed on the StepOnePlus Real-Time PCR Systems (Thermo, Waltham, MA, United States) with TB Green^®^ Premix Ex Taq^TM^ II (RR820Q, Takara, Beijing, China). For qRT-PCR run, the following protocol was followed: 95°C for 5 min, followed by 40 cycles of 95°C for 10 s, 60°C for 30 s. Human or mouse *Hprt* gene was used as a reference gene for relative quantification. Primer sequences are listed in Supplementary Table 1. The virus stocks, the supernatants of the cells, the SnPPIX-ZIKV mixtures or the peptide-ZIKV mixtures were applied for plaque forming assay to determine the virus titer as previously described (Vicenti et al., 2018). Briefly, Vero cells were seeded on 12-well plates (2.5 × 10^5^ cells/well) before incubation with samples. The diluted supernatants or peptide-ZIKV mixtures were incubated with Vero cells for 2 h at 37°C and washed with DMEM. These cells were cultured in solid DMEM containing 1% low melting agarose and 2% FBS for 6 days in 5% CO_2_ at 37°C. The plaques were counted after fixing and crystal violet staining.

### Immunoblot Analysis

Anti-SOCS1 (3950S, CST, Danvers, MA, United States), anti-SOCS3 (52113, CST, Danvers, MA, United States), anti-p-Stat1 (9167, CST, Danvers, MA, United States), anti-Stat1 (9172, CST, Danvers, MA, United States) and anti-β-actin (3700S, CST, Danvers, MA, United States) were used in this study. The secondary antibodies are HRP-labeled anti-rabbit and anti-mouse antibodies (CST, Danvers, MA, United States). The total proteins were separated by 12% sodium dodecyl sulfate polyacrylamide gel electrophoresis (SDS-PAGE) and electro-transferred onto a polyvinylidene difluoride (PVDF) membrane (Roche, Germany). The PVDF membrane was blocked with TBST (2.42 g/L Tris base, 8 g/L NaCl, 0.1% Tween 20, pH 7.6) containing 5% non-fat dried milk (BD, Franklin Lakes, NJ, United States) at room temperature for 2 h. After washing three times with TBST, the membrane was incubated overnight with primary antibodies at 4°C, and incubated with the secondary antibody for 1 h at room temperature. After washing with TBST, the membrane was developed with an enhanced chemiluminescence kit (TIANGEN, Beijing China) in a dark room. The intensity of signaling was quantified by ImageJ 1.8.0 software (National Institutes of Health, United States).

### Mouse Experiments

To test the toxicity of ZY13 *in vivo*, one-day-old Balb/c mouse pups were injected intraperitoneally with 40 mg/kg of ZY13 daily and monitored for 3 weeks. As previously reported, one-day-old Balb/c mouse pups were injected intraperitoneally with 50 μl 0.9% NaCl solution with or without 10^5^ PFU of ZIKV one hour before treatment (Wu et al., 2018). Mice were then intraperitoneally injected 5 mg/kg or 20 mg/kg of ZY13, 20 mg/kg of Scr-ZY13, 30 mg/kg of AQ or an equal volume of the solvent per day following the infection. The body weights of the mice were monitored for 14 days post infection. Mice were euthanized at day 14 post infection, and major organs, especially the brain and the spinal cord, were collected for further analysis.

For hematoxylin and eosin (H&E) staining, half of the brains of mice were fixed in 10% formalin and 5 μm sections were stained and examined by microscope. Selected slides were scored into five degrees on a scale from Grade 0 to Grade 4 (based on the pathological changes such as focal necrosis and immune cell invasion: 0, none; 1, subtle; 2, mild; 3, moderate; 4, marked) by two pathologists independently. The wet tissues were weighted and homogenized in PBS for plaque-forming assays as described above. Mouse experiments were conducted in accordance with the guidelines and were approved by the Animal Care and Use Committee, Kunming Institute of Zoology, Chinese Academy of Sciences (SMKX-20190513-01).

### Statistical Analysis

Figures were generated using the GraphPad Prism 6 software (Version 6.01, GraphPad Software, Inc., San Diego, CA, 2012, United States). Data are given as mean ± SEM. Statistical analysis was performed using two-tailed Student *t*-test or two-way ANOVA with multiple comparison correction. *P*-value ≤ 0.05 was considered significant.

## Results

### ZY13 Inhibits ZIKV Infection *in vitro*

**FIGURE 1:**
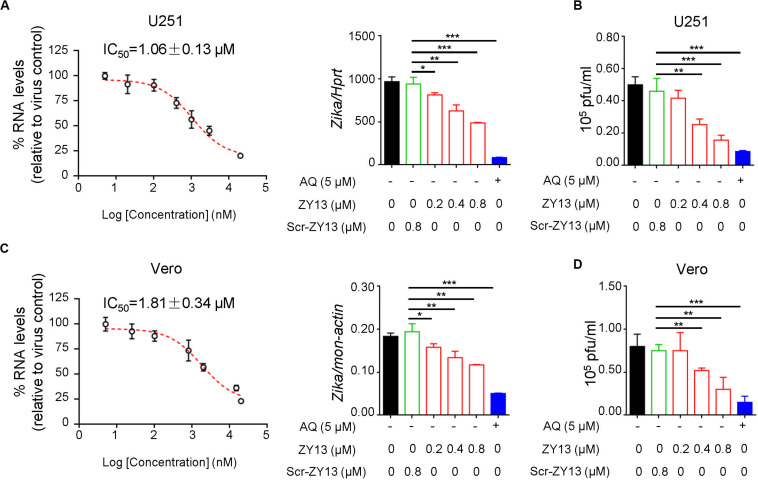
ZY13 inhibits ZIKV infection *in vitro*. **(A)** qPCR analysis of ZIKV in U251 cells after 24 h infection with ZIKV (MOI = 1) alone or in combination with ZY13, Scr-ZY13 and 5 μM of Amodiaquine (AQ). The IC_50_ of ZY13 against ZIKV infection is 1.06 ± 0.13 μM in U251 cells. Results are presented relative to those of human *Hprt* and compared to Scr-ZY13 group (negative control). **(B)** ZIKV titration in the supernatants of U251 cells after infection with ZIKV (MOI = 1) alone or in combination with ZY13, Scr-ZY13 and 5 μM of Amodiaquine (AQ). ZY13 administration at a concentration of 0.8 μM significantly reduced the production of infectious virions of ZIKV. **(C)** qPCR analysis of ZIKV in Vero cells after infection with ZIKV (MOI = 0.5) alone or in combination with ZY13, Scr-ZY13 and 5 μM of Amodiaquine (AQ). The IC_50_ of ZY13 against ZIKV infection is 1.81 ± 0.34 μM in Vero cells. Results are presented relative to those of monkey *Hprt* and compared to Scr-ZY13 group (negative control). **(D)** ZIKV titration in the supernatants of Vero cells after infection with ZIKV (MOI = 0.5) alone or in combination with ZY13, Scr-ZY13 and 5 μM of Amodiaquine (AQ). ZY13 administration at a concentration of 0.8 μM significantly reduced the production of infectious virions of ZIKV. Data represent 3 independent experiments and are presented as mean ± SEM. *P < 0.05; **P < 0.01; ***P < 0.001.

To determine the antiviral activity of ZY13 against ZIKV infection, we analyzed the replication of ZIKV by qRT-PCR at 24 h post infection. We found that ZY13 inhibited ZIKV infection in U251 cells in a dose-dependent manner with 50% inhibitory concentration (IC_50_) of 1.06 ± 0.13 μM (Figure 1A). In line with these results, plaque forming assay also showed that ZY13 administration significantly reduced the virion production (Figure 1B). It should be noted that ZY13 administration at a concentration of 0.8 μM significantly reduced the ZIKV infection (approximately 50%) in U251 rather than its scrambled control peptide Scr-ZY13 (less than 10%) (Figures 1A,B). We also used the Vero cells to test the anti-ZIKV activity of ZY13. As shown in Figures 1C,D, ZY13 showed remarkable inhibitory effect on the replication (IC_50_ = 1.81 ± 0.34 μM) and virion production of ZIKV in Vero cells. No effect on cell viability was observed for the used peptide concentrations (Supplementary Figures S1A,B). These results suggest that ZY13 can inhibit ZIKV infection *in vitro* and may interact with the viral particle or other factors. To determine whether ZY13 can inhibit other flavivirus infection, we tested the antiviral activity of ZY13 on DENV-2. As shown in Supplementary Figure S2, ZY13 showed weakened effect on the replication of DENV-2 comparing to ZIKV.

### ZY13 Exerts Antiviral Activity Through a Direct Effect on ZIKV Virion

**FIGURE 2:**
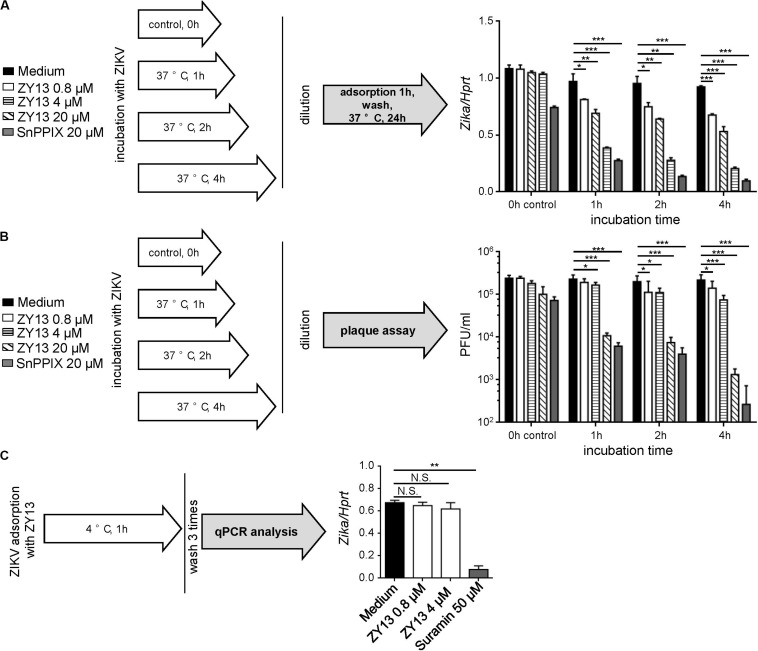
ZY13 directly inactivates ZIKV virions. **(A)** A schematic diagram showing the experimental process (left). To determine the ZY13 mediated ZIKV inactivation, peptides at various concentration were added to ZIKV (1 × 10^6^ PFU/ml) followed by incubation at 37°C for 1, 2, and 4 h. After incubation, the mixtures were diluted 200 times to keep the final concentration of the compounds below 0.1 μM. qPCR analysis of ZIKV genes in U251 cells after infection with ZIKV (diluted 200 times, about 0.05 MOI) pre-incubated with or without ZY13 and SnPPIX at the indicated time and concentrations. ZIKV pre-incubation with 20 μM ZY13 for 4 h significant decreases in about 70% the infectivity of ZIKV (right). Results are presented relative to those of human *Hprt*. **(B)** A schematic diagram showing the experimental process (left). Assays were performed as described in **(A)**. The diluted supernatants or peptide-ZIKV mixtures were applied for plaque forming assay immediately. **(C)** A schematic diagram showing the experimental process (left). Assays were performed by pre-incubating U251 cells with ZIKV (MOI = 1), ZIKV + peptides or ZIKV + Suramin at 4°C for 1 h. The attached ZIKV were detected by qRT-PCR after washing the cells three times with DMEM. Data represent 3 independent experiments and are presented as mean ± SEM. *P < 0.05; **P < 0.01; ***P < 0.001.

To further determine the antiviral mechanism of ZY13, we tested different treatments using the peptide. Interestingly, the results show that pre-incubation with ZY13 decreases the infectivity of ZIKV (Figure 2A). According to the results shown in Figure 2A, pre-incubation with 20 μM ZY13 for 4 h led to a significant decrease in infectivity of ZIKV. Following this, we performed viral inactivation assays to investigate whether ZY13 could directly act on the ZIKV virions. We found that ZY13 pre-incubation inactivates ZIKV particles and leads to remarkable lower plaque forming units (Figure 2B). ZIKV pre-incubation with 20 μM of ZY13 for 1, 2, and 4 h significantly reduced over 95% of the plaque forming units whereas pre-incubation with 0.8 or 4 μM of ZY13 were not that obvious. Next, we investigated whether ZY13 treatment inhibits the initial attachment of ZIKV according to the scheme shown in Figure 2C. The results indicated that ZY13 did not affect the virus attachment even at the relatively high concentration of 4 μM (Figure 2C). It is reported that a certain number of antivirals can directly inactivate the viral particle (He et al., 2018). These data suggested that ZY13 exerts anti-ZIKV activity by directly inactivating ZIKV infectious virions. Meanwhile, the different antiviral efficacy shown in Figures 1, 2 indicated that other antiviral mechanisms of ZY13 may also exist.

### ZY13 Downregulates the Expression of Axl Post ZIKV Infection

**FIGURE 3:**
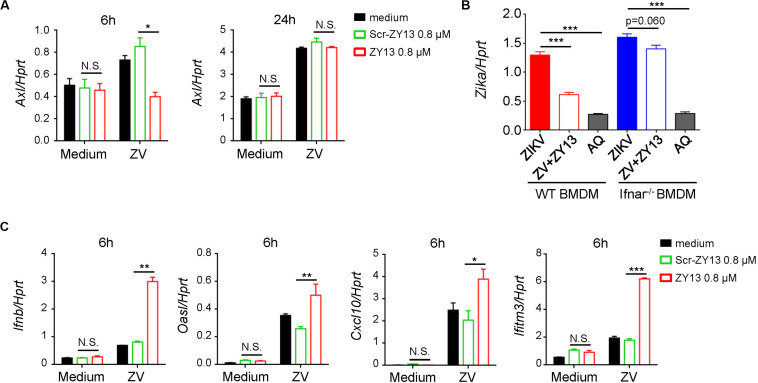
ZY13 regulates the expression of *Axl* and ISGs during ZIKV infection. **(A)** qPCR analysis of *Axl* genes in U251 cells after infection with ZIKV (MOI = 1) alone or in combination with ZY13 at indicated time points; results are presented relative to those of human *Hprt*. **(B)** qPCR analysisof ZIKV in BMDM cells after 24 h infection. ZY13 (0.8 μM) but not AQ (5 μM) exhibited more notable antiviral effects in WT BMDM cells than *Ifnar^–/–^* BMDM cells. **(C)** qPCR analysis of type I IFN-*Ifnb*, and its inducible genes *Oasl, Cxcl10*, and *Ifitm3* at 6 h post ZIKV infection. Data represent 3 independent experiments and are presented as mean ± SEM. *P < 0.05; **P < 0.01; ***P < 0.001, N.S., not significant.

According to previous studies, AXL was considered as a candidate entry factor for ZIKV infection (Govero et al., 2016; Ma et al., 2016; Nowakowski et al., 2016). However, recent studies argue against the role of AXL as an entry receptor (Wells et al., 2016; Chen et al., 2018). Instead, it was reported that the presence of AXL attenuated the ZIKV activated type I interferon (IFN) antiviral signaling (Chen et al., 2018). In brief, the AXL receptor is identified as ZIKV entry receptor and/or antiviral immune modulator and may play an important role during ZIKV infection in a cell-type-specific manner (Strange et al., 2019). In order to elucidate the mechanism by which ZY13 suppresses ZIKV infection, we analyzed the expression of relevant genes with or without ZY13 administration in U251 cells post ZIKV infection. We found that ZY13 significantly reduced the expression level of *Axl* at 6 h post ZIKV infection but not at 24 h in U251 cells (Figure 3A). The results indicated that ZY13 may act on the AXL related type I IFN signaling.

### ZY13 Activates Interferon Signaling and Antiviral Gene Expression During ZIKV Infection

Interferon signaling plays a key role in restricting ZIKV infection and cells lacking IFN-α/β are more permissive to infection with ZIKV and other flaviviruses. Interestingly, we found that ZY13 exhibited different antiviral effects compared with AQ in BMDM cells from WT mice and *Ifnar^–/–^* mice (Figure 3B). In BMDM cells from WT mice, 0.8 μM of ZY13 significantly decrease the infectivity of ZIKV. However, the same dose of ZY13 administration was less effective in BMDM cells from *Ifnar^–/–^* mice. The results shown in Figure 3B indicated that the antiviral activity of ZY13 may rely on the presence of type I IFN. As it was reported that AXL can attenuate type I IFN antiviral signaling (Chen et al., 2018), we, therefore, hypothesized that inhibition of the expression of *Axl* after ZY13 treatment may enhance the host type I IFN signaling. As anticipated, we found that ZY13 administration induced the expression of type I IFN-*Ifnb*, and its inducible genes such as *Oasl, Cxcl10*, and *Ifitm3* at 6 hours post ZIKV infection (Figure 3C). These results suggested that ZY13 can strengthen the host antiviral immune responses shortly post-ZIKV infection.

### ZY13 Suppresses the Expression of SOCS Family

**FIGURE 4:**
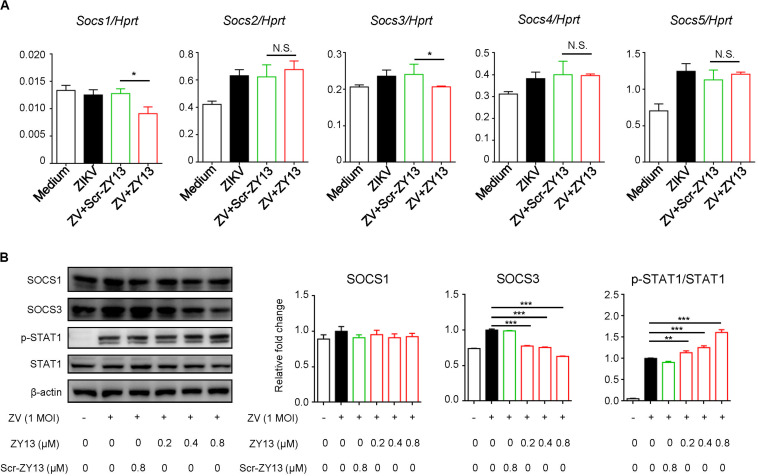
ZY13 suppresses the ZIKV induced expression of *Socs1* and *Socs3* at early phase of infection. **(A)** Cells were infected with ZIKV at 1 MOI with or without ZY13 administration. Expression analysis of *Socs1-5* genes in U251 cells with ZIKV alone or in combination with ZY13 were performed at 6 h post infection; results are presented relative to those of human *Hprt*. **(B)** Immunoblot analysis of phosphorylation of STAT1 and protein level of SOCS1, SOCS3 and STAT1 at 12 h post ZIKV infection (MOI = 1). β-actin was used as the loading control. The intensity of signaling was quantified (right). The Scr-ZY13 at a concentration of 0.8 μM was added as control. Data represent 3 independent experiments and are presented as mean ± SEM. *P < 0.05; **P < 0.01; ***P < 0.001; N.S., not significant.

Several studies have shown that AXL inhibits IFNAR signaling through the suppressor of cytokine signaling protein 1 (SOCS1) in a negative regulation manner (Rothlin et al., 2007; Chen et al., 2018). To further examine whether SOCS proteins play roles in ZY13-mediated induction of type I IFN signaling during ZIKV infection, we first analyzed the expression of *Socs* genes. As shown in Figure 4A, the expression of *Socs1* and *Socs3* were significantly reduced after ZY13 administration but not that of *Socs2*, *Socs4* and *Socs5*. Furthermore, immunoblot analysis showed that the protein level of SOCS3 was significantly reduced whereas the phosphorylation of STAT1 was increased with ZY13 co-administration (Figure 4B). Additionally, the protein level of SOCS3 and the phosphorylation of STAT1 were not significantly altered with Scr-ZY13 co-administration (Figure 4B). The expression of *Socs1* and *Socs3* were analyzed at 12 hours post infection and only the expression of *Socs3* was reduced after ZY13 administration (Supplementary Figure S3). These findings collectively suggest that ZY13 interacts with the AXL-SOCS negative regulation pathway of type I IFN signaling during ZIKV infection and thus enhancing the host’s immune response to ZIKV infection.

### ZY13 Has Antiviral Effects *in vivo*

**FIGURE 5:**
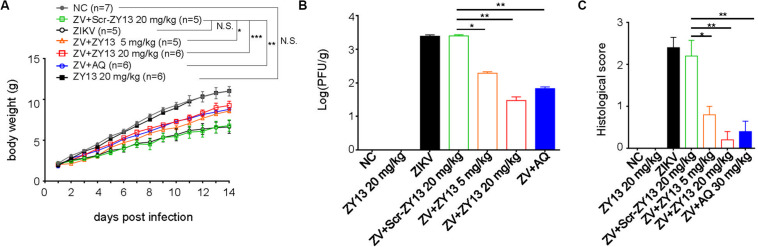
ZY13 restricts ZIKV infection in mouse pups. **(A)** One-day-old Balb/c mouse pups were intraperitoneally injected with 5 mg/kg, 20 mg/kg of ZY13, 20 mg/kg of Scr-ZY13, 30 mg/kg of AQ or an equal volume of the solvent per day following ZIKV infection. The body weights of the mice were monitored for 14 days post infection. **(B)** About a quarter of the brains of the mice at 14 days post infection were weighted before being homogenized in PBS for plaque-forming assays. ZY13 treatment reduces approximately90% (5 mg/kg) to 99% (20 mg/kg) infective ZIKV virions production in the mouse brain. **(C)** The pathological changes of the mouse brain were evaluated by histological scoring after H&E staining. Data represent 2 independent experiments and are presented as mean ± SEM. *P < 0.05; **P < 0.01; ***P < 0.001; N.S., not significant.

Despite some immunodeficient ZIKV mouse models, an immunocompetent mouse model of ZIKV infection is more appropriate for evaluating antiviral drugs (Gorman et al., 2018). And as has been well documented, ZIKV infection of 1-day-old mouse pups may be similar to that later in pregnancy (Wu et al., 2018). More importantly, no toxicity was observed in 1-day-old Balb/c mouse pups when injected intraperitoneally with 40 mg/kg of ZY13 daily. Thus, we used ZIKV infected mouse pups to further evaluate the *in vivo* antiviral effects of peptide ZY13. As shown in Figure 5A, treatment with ZY13 and AQ improved the ZIKV affected body growth. To further assess the anti-ZIKV efficiency of ZY13 *in vivo*, we analyzed the infectious viral titers in the main organs of the mice. Of note, the viral burdens in the brains were significantly lower after ZY13 and AQ treatment at 14 days post infection (Figure 5B). Additionally, there were fewer focal necrosis and immune cell invasion in the brain sections of ZY13 treated group than of ZIKV group at 14 days post infection as quantified by histological scoring (Figure 5C). Taken together, these results suggest that ZY13 can restrict ZIKV infection and improve the growth defects of ZIKV-infected mice.

## Discussion

The epidemic potential of mosquitoes transmitted ZIKV likely increased epidemic and infects tens of thousands of people (Baud et al., 2017; Yadav et al., 2019). More seriously, a recent study uncovered a hidden outbreak in Cuba during the year 2017 and highlights that ZIKV may still be silently spreading worldwide (Grubaugh et al., 2019). Currently, no effective vaccines or antiviral drugs have been approved for ZIKV. Venom systems have been used for predation and defense and venom-derived peptides have become an important source of antivirals (Cecilio et al., 2013; Hong et al., 2014; Holthausen et al., 2017; Ji et al., 2019). For example, a representative defensin-like antiviral peptide BmKDfsin4 from the scorpion *Mesobuthus martensii* Karsch was reported to inhibit Hepatitis B virus replication (Zeng et al., 2016). However, natural antiviral peptides sometimes have some drawbacks for their further clinical use (Boto et al., 2018). Therefore, developing natural peptides derived analogs with improved pharmacological properties and reduced side effects is imperative (Fjell et al., 2011; Jin et al., 2016; Boto et al., 2018).

Our previous work established that BF-30 and its analogs show effective broad-spectrum antimicrobial abilities and therapeutic potential for pancreatic cancer (Wang et al., 2011; Jin et al., 2016; Fang et al., 2019; Xu C. et al., 2019). In this study, we found that ZY13 exhibits remarkable anti-ZIKV efficacy *in vitro* with IC_50_ of 1.06 ± 0.13 μM in U251 cells and IC_50_ of 1.81 ± 0.34 μM in Vero cells. Meanwhile, ZY13 shows relatively weaker effect on the replication of DENV-2. The LL-37 cathelicidin-derived peptides were reported to directly inactivate ZIKV (He et al., 2018). It is reported that BF-30 can effectively inhibit influenza virus and plays a role in the stage of virus invasion by causing viral particle membrane fusion (Xu J. et al., 2019). In our previous study, we found that ZY13 has an amphipathic helix structure and can cause the membrane perforation of *C. albicans* (Jin et al., 2016). Therefore, we reasonably speculated that ZY13 may interrupt the viral membrane integrity of the ZIKV virions. Firstly, considering ZIKV pre-incubation with 20 μM of ZY13 significantly reduced over 95% of the plaque forming units, ZY13 was revealed to play antiviral roles by inactivating the viral particles of ZIKV. Confusingly, we found that ZY13 exhibited more notable antiviral effects in WT BMDM cells than in *Ifnar^–/–^* BMDM cells (Figure 3B). Given this evidence, we further explored other antiviral mechanisms of ZY13 involved in inhibiting ZIKV infection. Remarkably, we found that ZY13 also possesses immunomodulatory functions by modulating type I IFN signaling. Type I IFN and the genes it stimulates are key mediators in controlling ZIKV infection and replication (Barrows et al., 2018). Recently, AXL-SOCS1 have been identified as negative regulators of type I IFN signaling and have been shown to play an important role during ZIKV infection (Strange et al., 2019). Our result suggests that ZY13 administration downregulates the expression of *Axl*, *SOCS1* and *SOCS3* at early stage post ZIKV infection. Consistent with this observation, ZY13 administration was associated with an increase in the expression of type I IFN inducible genes *Oasl, Cxcl10*, and *Ifitm3* at 6 h post ZIKV infection. The ZY13 induced expression of type I IFN inducible genes may favor the elimination of ZIKV during the early phase of infection. Since AXL has closer links to ZIKV rather than other flaviviruses, these findings may partially explain the different antiviral efficacy of ZY13 against ZIKV and DENV-2.

ZIKV infection can result in cortical thinning and neurodevelopment anomalies of fetus. It is possible and necessary to evaluate the *in vivo* antiviral efficiency of ZY13 by using an appropriate mouse model (Wu et al., 2018). In this study, we demonstrated that ZY13 could restrict the viral load in the brain and improve the growth defects of ZIKV-infected mouse pups. The development of the brain of rodents is immature at birth. In this sense, 1 day old neonate mice brain development may well correspond to that seen in the later stages of human pregnancy (Wu et al., 2018). On the basis of the results, it is reasonable to say that ZY13 may have therapeutic potential *in vivo*. However, a study has shown that different ZIKV strains respond differently to the natural compound silvestrol (Elgner et al., 2018). Thus, for further anti-ZIKV agent development of ZY13, more experiments should be carried out both *in vitro* and *in vivo* by using different ZIKV genotypes.

It is also worth mentioning that a growing body of evidence indicates that antimicrobial peptides may participate in the transcriptional regulation of bacteria and mammal cells (Velarde et al., 2014; Munoz et al., 2016). Moreover, the scrambled control peptide Scr-ZY13 shows little antiviral and immunomodulatory activity during ZIKV infection. Thus, as a reasonable conjecture, it is likely that ZY13 may act as a regulator of gene transcription during ZIKV infection and therefore warrants further studies.

**FIGURE 6:**
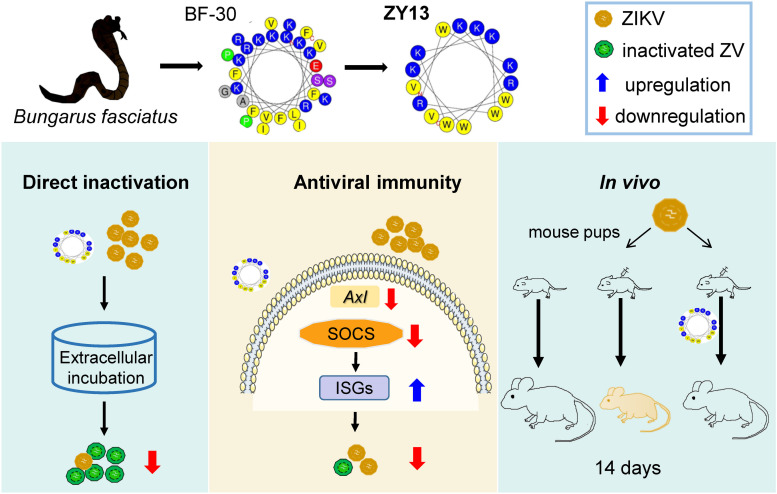
Overview of the main findings in this study. Snake venom derived peptide ZY13 can directly inactivate ZIKV and strengthen the host antiviral immunity via AXL-SOCS pathway. The *in vivo* efficiently of ZY13 was further confirmed in ZIKV-infected mouse pups.

Recently, the disclosure of ZIKV silent spread has underscored its latent threat to public health (Grubaugh et al., 2019). Considering there are still no approved vaccines, the development of specific drugs for ZIKV infection is paramount. The promising results observed in our present study suggest that ZY13, a representative analog of snake cathelicidin BF-30, is a promising molecule against ZIKV infection (summarized in Figure 6). Furthermore, our study also highlights the importance of venom peptides as prototypes for antivirals development.

## Data Availability Statement

The datasets generated for this study are available on request to the corresponding author.

## Ethics Statement

The animal study was reviewed and approved by the Animal Care and Use Committee, Kunming Institute of Zoology, Chinese Academy of Sciences.

## Author Contributions

MX, MJ, JH, TZ, YC, XB, GM, and LJ conducted the experiments. RL and LJ designed the experiments, provided the guidance for the research, analyzed the data, and wrote the manuscript. XB and JM revised the manuscript. All the authors read and approved the contents of the manuscript.

## Conflict of Interest

The authors declare that the research was conducted in the absence of any commercial or financial relationships that could be construed as a potential conflict of interest.
