# Supplementary material for: Snake Cathelicidin Derived Peptide Inhibits Zika Virus Infection
Source: Front Microbiol. 2020 Aug 4;11:1871. doi: 10.3389/fmicb.2020.01871 (PMC7417475; doi:10.3389/fmicb.2020.01871)
Supplement: Supplementary file 1 [file Data_Sheet_1.pdf]

Supplementary Material

1 Supplementary tables

Supplementary Table 1. Real Time qPCR Primer Sequences

Gene	5' primer	3'primer
Socs1	*GGTCCCCCTGGTTGTTGTA*	*TAGGAGGTGCGAGTTCAGGT*
Socs2	*TTAAAAGAGGCACCAGAAGGAAC*	*AGTCGATCAGATGAACCACACT*
Socs3	*CCTGCGCCTCAAGACCTTC*	*GTCACTGCGCTCCAGTAGAA*
Socs4	*GCCGACAGAAAAGACGGTTAT*	*GGTTCCTTAAAGACACTTCGGTT*
Socs5	*AGAGATTCCTACTCTCGACATGC*	*GCCCACAGTATCCTGCAACC*
hHprt	*GCTATAAATTCTTTGCTGACCTGC TG*	*AATTACTTTTATGTCCCCTGTTGACT GG*
mHprt	*CTCATGGACTGATTATGGACAGGA C*	*GCAGGTCAGCAAAGAACTTATAGCC*
mon-Actin	*CTACAATGAGCTGCGTGTGG*	*GTACATGGCTGGGGTGTTGA*
Zika	*GACGCCAGAGTTTGTTCAGA*	*TGGCTTCCTGGAATCTCTCT*
Denv2	*CAGGCTATGGCACYGTCACGA*	*CCATYTGCAGCARCACCATCTC*
hAxl	*CAGAGCCCGTGGACCTACT*	*TTCGCAGGAGAAAGAGGATG*
hIfnb	*AGCTGAAGCAGTTCCAGAAG*	*AGTCTCATTCCAGCCAGTGC*
hCxcl10	*GTGGCATTCAAGGAGTACCTC*	*TGATGGCCTTCGATTCTGGATT*
hOasl	*CTGATGCAGGAACTGTATAGCAC*	*CACAGCGTCTAGCACCTCTT*
Ifitm3	*CATCCTCATGACCATTCTGC*	*TCAGTGATGCCTCCTGATCT*

2 Supplementary Figures

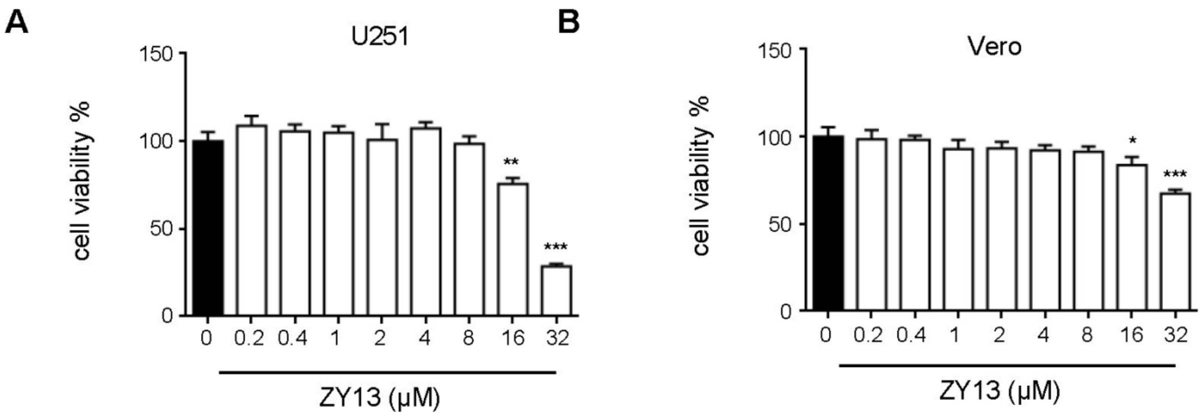

Supplementary Figure 1. ZY13 inhibits ZIKV infection in vitro (related to **Figure 1**). **(A)** The cytotoxicity of ZY13 on U251 cells. **(B)** The cytotoxicity of ZY13 on Vero cells. Data represent at least 2 independent experiments and are presented as mean ± SEM. *, $P < 0.05$; **, $P < 0.01$; ***, $P < 0.001$.

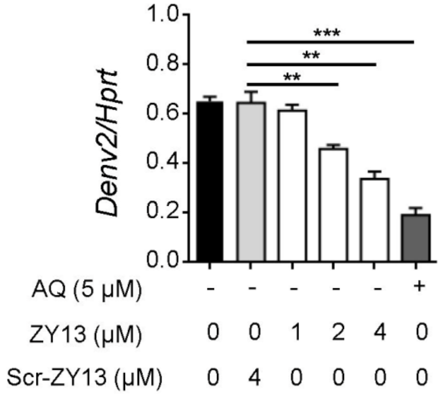

Supplementary Figure 2. ZY13 shows antiviral effect on the replication of DENV-2. Cells were infected with DENV-2 at 1 MOI with or without ZY13 and AQ administration. Data represent 3 independent experiments and are presented as mean ± SEM. **, $P < 0.01$; ***, $P < 0.001$.

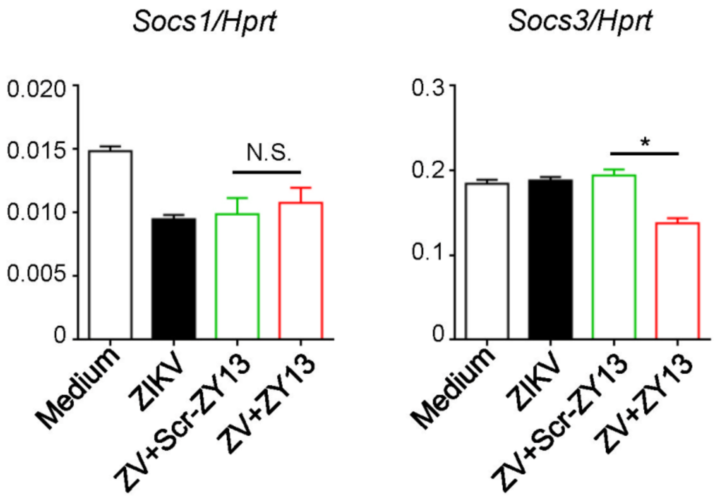
Socs1/Hprt
0.020
0.015
0.010
0.005
0
N.S.
Medium
ZIKV
ZV+Scr-ZY13
ZV+ZY13
Socs3/Hprt
0.3
0.2
0.1
0
*
Medium
ZIKV
ZV+Scr-ZY13
ZV+ZY13

Supplementary Figure 3. The expression of *Socs1* and *Socs3* at 12 hours post ZIKV infection**.** Data represent 3 independent experiments and are presented as mean ± SEM. *, $P < 0.05$, N.S., Not Significant.

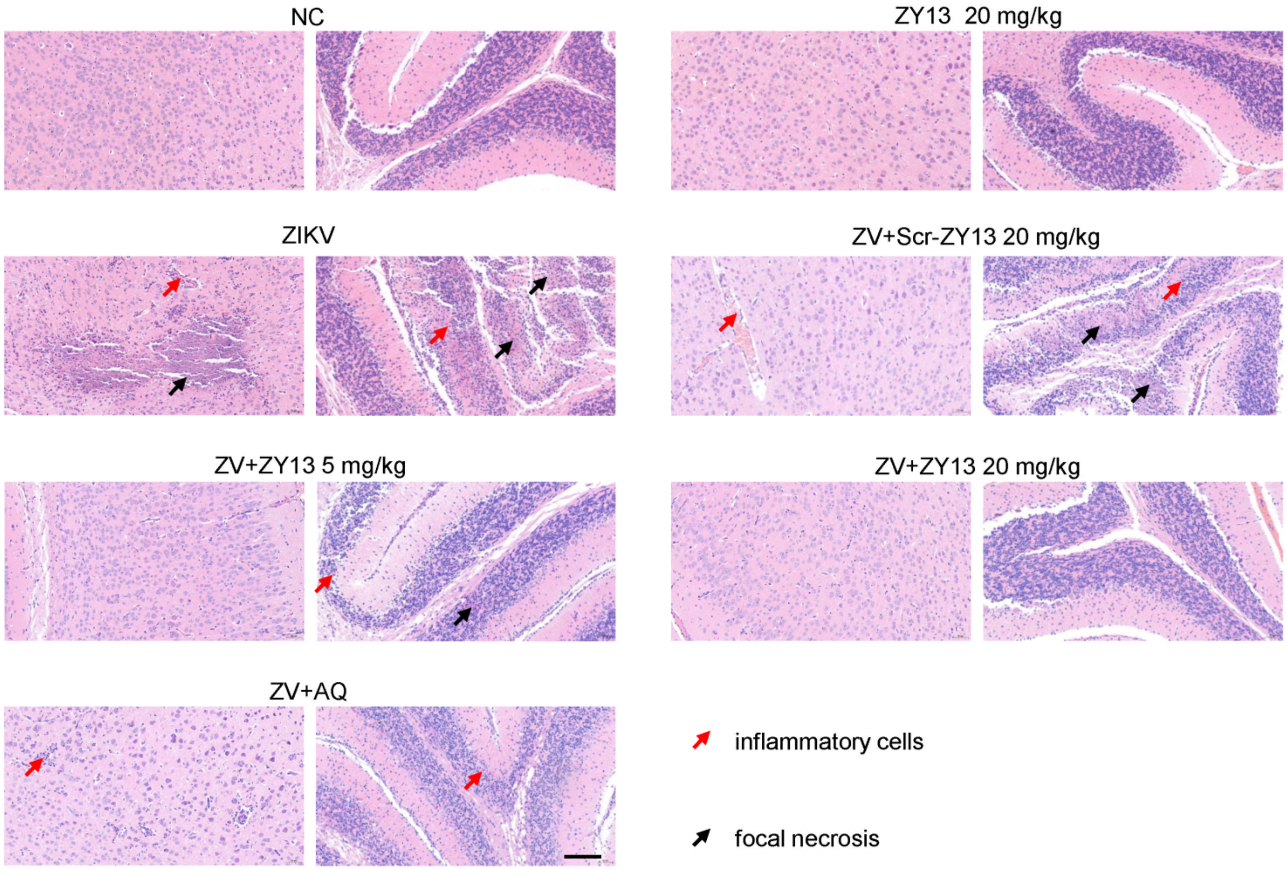

Supplementary Figure 4. H&E staining of the mouse brain (related to **Figure 5C**). Half of the brains of mice were fixed in 10% formalin and 5 μm sections were stained and examined by microscope. Typical inflammatory cells infiltration and focal necrosis were indicated by arrows. Scale bar, 120 μm.
